# Functional measures, inflammatory markers and endothelin-1 as predictors of 360-day survival in centenarians

**DOI:** 10.1007/s11357-015-9822-9

**Published:** 2015-08-21

**Authors:** Jan Szewieczek, Tomasz Francuz, Jan Dulawa, Katarzyna Legierska, Beata Hornik, Iwona Włodarczyk, Magdalena Janusz-Jenczeń, Agnieszka Batko-Szwaczka

**Affiliations:** 1 Department of Geriatrics, School of Health Sciences in Katowice, SUM, SPSK NR 7 SUM GCM, ul. Ziolowa 45/47, 40-635 Katowice, Poland; 2 Department of Biochemistry, School of Medicine in Katowice, SUM, Katowice, Poland; 3 Department of Internal Medicine and Metabolic Diseases, School of Health Sciences in Katowice, SUM, Katowice, Poland; 4 Department of Internal Nursing, School of Health Sciences in Katowice, SUM, Katowice, Poland

**Keywords:** Centenarians, Mini-Mental State Examination, Activities of daily living, 360-day survival, Interleukin-6, Endothelin-1

## Abstract

Centenarians represent a rapidly growing population. To better characterize this specific age group, we have performed a cross-sectional study to observe associations between functional measures and a range of biochemical markers, including inflammatory markers and their significance as predictors of 360-day survival. Medical history and physical and functional assessment (Mini-Mental State Examination (MMSE), Katz Index (activities of daily living, ADL) and Barthel Index (Barthel Index) of Activities of Daily Living, and Lawton Instrumental Activities of Daily Living Scale (Lawton IADL)) were conducted on 86 101.9 ± 1.2-year-old (mean ± SD) subjects (70 women, 16 men). Blood tests were performed on 84 subjects of whom 43 also had extended biomarker assessment. As a reference group 30 51.8 ± 5.0-year old healthy subjects (20 women, 10 men) were recruited. The centenarians received follow-up phone calls. Fifty-two centenarians (60 %) survived ≥360 days. Longer survival was associated with higher MMSE (hazard ratio, HR = 0.934, 95 % confidence interval (CI) 0.896–0.975, *P* = .002), ADL (HR = 0.840, 95 % CI 0.716–0.985, *P* = .032), Barthel Index (HR = 0.988, 95 % CI 0.977–0.999, *P* = .026), and albumin level (HR .926, 95 % CI 0.870–0.986, *P* .016) and with lower white blood cell (WBC) (HR = 1.161, 95 % CI 1.059–1.273, *P* = .001), C-reactive protein (CRP) (HR = 1.032, 95 % CI 1.014–1.050, *P* < .001), IL-6 (HR = 1.182, 95 % CI 1.047–1.335, *P* = .007), and endothelin-1 (ET-1) level (HR = 3.711, 95 % CI 1.233–11.169, *P* = .020). Centenarians had higher 360-day survival probability with MMSE ≥13 (*P* < .001), ADL ≥1 (*P* < .001), Barthel Index ≥15 (*P* < .001), Lawton IADL ≥10 points (*P* = .009), WBC <8.3 G/L (*P* = .039), CRP <10 mg/L (*P* < .001), IL-6 <6 pg/mL (*P* .002), and ET-1 <1.1 pg/mL (*P* .007). Our results indicate that functional measures, inflammatory markers, and endothelin-1 are predictors of 360-day survival in centenarians.

## Background

Survival over 100 years of age used to be an extraordinary occurrence. However, over the past several decades, the world population of the oldest old is growing rapidly. According to the Central Statistical Office of Poland, the number of centenarians per 100,000 people will increase from 1 in 1970 to 9 in 2010 and to a projected 174 in the year 2050 (Central Statistical Office of Poland 2014). Clinical studies concerning centenarians are critical for equipping policy makers, social care providers, and clinicians with appropriate knowledge regarding the health and social needs of this unique age group. There is increasing evidence that would suggest changes in the meaning of health indicators as human life progresses, making centenarians distinctive even of younger seniors. A pervasive feature of aging is low-grade, chronic, systemic inflammation (inflammaging) revealed by elevated levels of inflammatory biomarkers such as C-reactive protein, interleukin-6, and tumor necrosis factor alpha (Maes et al. 1999; Franceschi et al. 2000; Bruunsgaard et al. 2003; Michaud et al. 2013). This state of mild inflammation is predictive of numerous aging phenotypes and represents a highly significant risk factor for both morbidity and mortality among the elderly (Franceschi et al. 2000; Franceschi and Campisi 2014). Inflammation is strongly associated with incident cardiovascular events (Cesari et al. 2003) showing a close relationship between inflammation and vascular aging (Sun 2015). It has also been shown that inflammation is a key predictor of health status in the elderly (Barodka et al. 2011). Inflammaging may also be involved in the pathophysiology of neurodegenerative disorders and cognitive impairment (e.g. Alzheimer disease) (Tan et al. 2007; Michaud et al. 2013; Cunningham and Hennessy 2015). As is the case with unsuccessful aging, centenarians are also affected by systemic inflammation as well as other signs of age-related homeostasis disorders, e.g., decreased antioxidant levels and hypercoagulable states (Franceschi and Campisi 2014). However, as opposed to those who age poorly, centenarians survive or delay the onset of chronic age-related disease such as type 2 diabetes, cardiovascular disease, severe dementia, and cancer. Centenarians have even been shown to “escape” chronic disease (Evert et al. 2003; Arnold et al. 2010; Sebastiani et al. 2013), which has come to be considered “the paradox of centenarians” (Franceschi and Campisi 2014). Despite being afflicted with clinically evident morbidities, there is evidence that centenarians appear to compress their disability toward the end of their lives (Terry et al. 2008). Andersen et al. (2012) observed a progressive delay in the age of onset of physical and cognitive function impairment, age-related diseases, and overall morbidity with increasing age. As the limit of the human life span was effectively approached with supercentenarians, compression of morbidity was generally observed. Healthy centenarians constitute a subpopulation of elderly that is characterized both by increased longevity and maintenance of functional independence (Franceschi and Bonafè 2003). Therefore, centenarian studies contribute substantially to understanding the aging process and the factors influencing longevity (Willcox et al. 2010). Relationships between inflammaging, vascular aging, functional status, and longevity have not been extensively explored for centenarians. We completed a cross-sectional study to characterize the health state of centenarians in Upper Silesia, the most industrialized region of Poland. To assess mental and physical performance, we used measures of cognitive function and independence in basic and instrumental activities of daily living used in comprehensive geriatric assessment. Among widely accepted inflammatory markers, in order to assess the inflammatory status of our study group, we analyzed the blood work for factors assessing immune system activation, extracellular matrix turnover, and vascular injury. We chose interleukin-6 (IL-6), tumor necrosis factor alpha (TNF-α), C-reactive protein (CRP), and CD40 ligand (CD40L) as widely accepted inflammatory markers. Activation of the immune system was assessed by examining levels of integrins intercellular adhesion molecule-1 (ICAM-1), vascular cell adhesion molecule-1 (VCAM-1), and P-selectin, which are induced by proinflammatory cytokines IL-6 and TNF-α and are responsible for inflammatory cell adhesion and penetration of the vascular wall. Extracellular matrix turnover was assessed by examining levels of metalloproteinases 1 and 9 and tissue inhibitor of metalloproteinases 1 (Thakore et al. 2007; Zakynthinos and Pappa 2009). To understand the vascular function of our study population, we assessed endothelin-1, a potent vasoconstrictor, and plasminogen activator inhibitor 1, an important risk factor for thrombosis and atherosclerosis (Paulus et al. 2011). Our results were presented partially in our preliminary papers, where we focused on the relationship between blood pressure, mental and physical performance, and 180-day survival in the study group (Szewieczek et al. 2011, 2015). In this paper, we present an analysis of associations between functional measures and a range of biochemical markers, including inflammatory markers, to assess their significance as predictors of 360-day survival.

## Methods

### Participants

Our study was conducted from January 2007 to August 2013 and included 86 consecutive subjects (70 women and 16 men) aged 100.9 ± 1.2 years (mean ± SD) in the range 99.1 to 104.7 years who responded positively to invitation letters (8 % of addressees). A residential visit by the team consisting of a physician and two nurses was scheduled with the subject and their caregiver. Selected biochemical markers were also determined in a control group of 30 healthy subjects (20 women and 10 men) aged 51.8 ± 5.0 years.

### Measurements

The assessment included a structured interview, physical examination, functional measures (Katz ADL, Barthel Index, Lawton IADL, and MMSE), and blood sampling. The Mini-Mental State Examination (MMSE) (Folstein et al. 1975) was used to assess global cognitive performance. The Katz Index of Independence in Activities of Daily Living (Katz ADL) (Katz et al. 1963), Barthel Index of Activities of Daily Living (Barthel Index) (Mahoney and Barthel 1965), and Lawton Instrumental Activities of Daily Living Scale (Lawton IADL) (Lawton and Brody 1969) were used to determine functional status. MMSE scores range from 0 to 30, Katz ADL scores from 0 to 6, Barthel Index scores from 0 to 100, and Lawton IADL scores from 9 to 27; higher scores indicate better functional state.

Laboratory tests performed for 84 centenarians (two subjects refused consent for blood sampling) are listed in Table 1. All biochemical markers were assessed in fasting blood samples.

**Table 1:** Clinical and functional characteristics of the centenarian study group with respect to 360-day survival (*n* = 86)

Measure	Survivors^a^ *n* = 52	Non-survivors^a^ *n* = 34	Survivors vs. non-survivors
	Mean ± SD or percentage		*P* value
Age, years	100.81 ± 0.99	101.09 ± 0.40	.581
Hypertension, %	94.2	76.5	.038
Diabetes mellitus, %	19.2	17.6	.854
Myocardial infarction in anamnesis, %	5.77	11.76	.555
Stroke in anamnesis, %	17.3	25.9	.704
Chronic obstructive pulmonary disease, %	13.5	14.7	.871
Chronic renal failure, %	19.0	17.6	.782
Cancer in anamnesis, %	3.85	9.68	.622
Ever smoking, %	8.33	25.9	.156
Number of used medications	4.83 ± 3.07	4.91 ± 3.18	.905
Body mass index, kg/m^2^	23.7 ± 4.9	23.7 ± 3.7	.940
Heart rate, beats per minute	73.6 ± 12.4	75.3 ± 12.2	.450
Systolic blood pressure, mmHg	153.4 ± 3.1	144.6 ± 24.8	.188
Diastolic blood pressure, mmHg	78.1 ± 16.5	76.2 ± 11.4	.436
Mean arterial pressure, mmHg	103.2 ± 18.3	99.0 ± 13.2	.310
Pulse pressure, mmHg	74.8 ± 26.3	68.1 ± 23.4	.389
MMSE score	17.9 ± 7.2	13.5 ± 8.7	.013
Katz ADL	3.52 ± 2.03	2.74 ± 2.30	.083
Barthel Index	62.6 ± 28.5	50.7 ± 35.9	.149
Lawton IADL	12.0 ± 3.6	11.2 ± 4.8	.069
Hemoglobin, mmol/L	7.89 ± 0.90	7.76 ± 0.81	.454
Red blood cells, T/L	4.29 ± 0.51	4.22 ± 0.52	.516
White blood cells, G/L	6.19 ± 2.06	7.95 ± 6.79	.063
Thrombocytes, G/L	227 ± 77	215 ± 86	.510
Bilirubin, μmol/L	12.1 ± 5.0	13.1 ± 8.6	.912
ALAT, nmol/L/s	366 ± 1057	202 ± 109	.349
Albumin, g/L	38.6 ± 5.4	35.9 ± 5.3	.033
Creatinine, μmol/L	96.5 ± 24.0	129.7 ± 166.5	.668
Cystatin C, mg/mL	1.72 ± 0.81	1.78 ± 0.79	.865
Glucose, mmol/L	5.32 ± 1.20	5.28 ± 0.87	.818
Insulin, pmol/L	62.2 ± 35.6	62.5 ± 4.9	.951
Thyrotropin, mIU/L	1.89 ± 1.39	1.89 ± 2.15	.552
Folate, nmol/L	13.6 ± 8.7	16.2 ± 1.2	.281
Vitamin B12, pmol/L	307 ± 242	249 ± 141	.463
Total cholesterol, mmol/L	5.13 ± 1.24	4.77 ± 1.26	.100
HDL cholesterol, mmol/L	1.65 ± 0.52	1.51 ± 0.58	.224
LDL cholesterol, mmol/L	2.96 ± 0.98	2.76 ± 1.12	.177
Triglycerides, mmol/L	1.17 ± 0.50	1.11 ± 0.44	.603
C-reactive protein, mg/L	6.83 ± 1.86	20.42 ± 41.14	.001

^a^Biochemical assays were completed for 84 subjects, among them 52 survivors and 32 non-survivors

In the course of the study, we extended the scope of assessed biomarkers for commonly used markers of endothelial dysfunction and vascular health. Thus, plasma levels of soluble P-selectin (sP-selectin), soluble intercellular adhesion molecule-1 (sICAM-1), soluble vascular cell adhesion molecule-1 (sVCAM-1), soluble CD40 ligand (sCD40L), interleukin-6 (IL-6), endothelin-1 (ET-1), TNF-α, plasminogen activator inhibitor-1 (PAI-1), matrix metalloproteinase-1 (MMP-1), matrix metalloproteinase-9 (MMP-9), and a tissue inhibitor of metalloproteinase (TIMP-1) were determined for 43 centenarians (28 survivors and 15 non-survivors) and 30 quinquagenarian healthy controls as a reference group. Commercially available ELISA kits (R&D Systems, Minneapolis, MN, USA) were used. Briefly, 5 mL EDTA-blood was centrifuged for 10 min at 1000 rpm; the obtained plasma was frozen in 1 mL aliquots at −80 °C until analysis. Just prior to sICAM-1, sVCAM-1, and sP-selectin determinations, samples were diluted 200 times so that they would remain within the dynamic range of the assay. sCD40L was determined in undiluted plasma samples. IL-6 and TNF-α were assessed using a high-sensitivity assay due to extremely low concentrations of the above molecules in human serum. The detection limit for IL-6 was 0.039 pg/mL, and for TNF-α was 0.106 pg/mL. ET-1 was detected using a chemiluminescent assay; the minimum detectable concentration was 0.064 pg/mL. A kit used for the assessment had about 50 % cross-reactivity with ET-2 and virtually no cross-reactivity with ET-3, big-ET-2/3, or other forms of endothelins. Absorbance and luminescence were read using Tecan Infinite M200 Pro Plate Reader (Tecan Group, Switzerland). Biomarker samples were reassayed at least twice and the mean value was used for analysis. The study subjects received follow-up phone calls for at least 360 days.

### Statistical Analysis

Data were analyzed using STATISTICA software version 10 (StatSoft, Inc., USA; StatSoft Poland). The Cox proportional hazards regression model was used for survival analysis. Nonparametric Mann–Whitney *U* test (for quantitative variables), chi-square test, *V*-square test, and Fisher’s exact test (for categorical variables) were used to compare centenarians who survived 360 days with those who did not survive. The Mann–Whitney *U* test was also used to compare selected biochemical markers (cytokines) among the groups of centenarians and healthy quinquagenarians. The Cox proportional hazards model was used for univariate and multivariate survival analysis. The Kaplan–Meier method was used to estimate survival probability in subgroups of centenarians with respect to selected variables, while differences between these subgroups were assessed with the Wilcoxon–Gehan statistic. Variables were tested to define the value corresponding with the lowest *P* level. The nonparametric Spearman’s rank correlation coefficient was used to assess relationships between blood pressure, functional measures, and variables analyzed in the study. *P* values <0.05 were considered statistically significant.

## Results

Hypertension (defined as systolic blood pressure ≥140 mmHg or diastolic blood pressure ≥90 mm or use of any antihypertensive drug) was present in 87 % of study subjects. A majority of subjects suffered from dementia (73 %), heart failure (65 %), and osteoarthritis (64 %). Sixty-three percent of subjects used at least one antihypertensive drug (among them beta blockers 14.0 %, ACEIs 29.1 %, ARBs 3.49 %, calcium blockers 12.8 %, spironolactone 15.1 %, thiazides 3.49 %, thiazide-like diuretics 8.14 %, loop diuretics 22.1 %), aspirin 23.3 %, GPIIb/IIIa inhibitors 3.49 %, statins 3.49 %, nitrates 18.6 %, insulin 2.33 %, oral antidiabetic drugs 6.98 %, and nonsteroidal anti-inflammatory drugs 8.14 %.

**Fig. 1:**
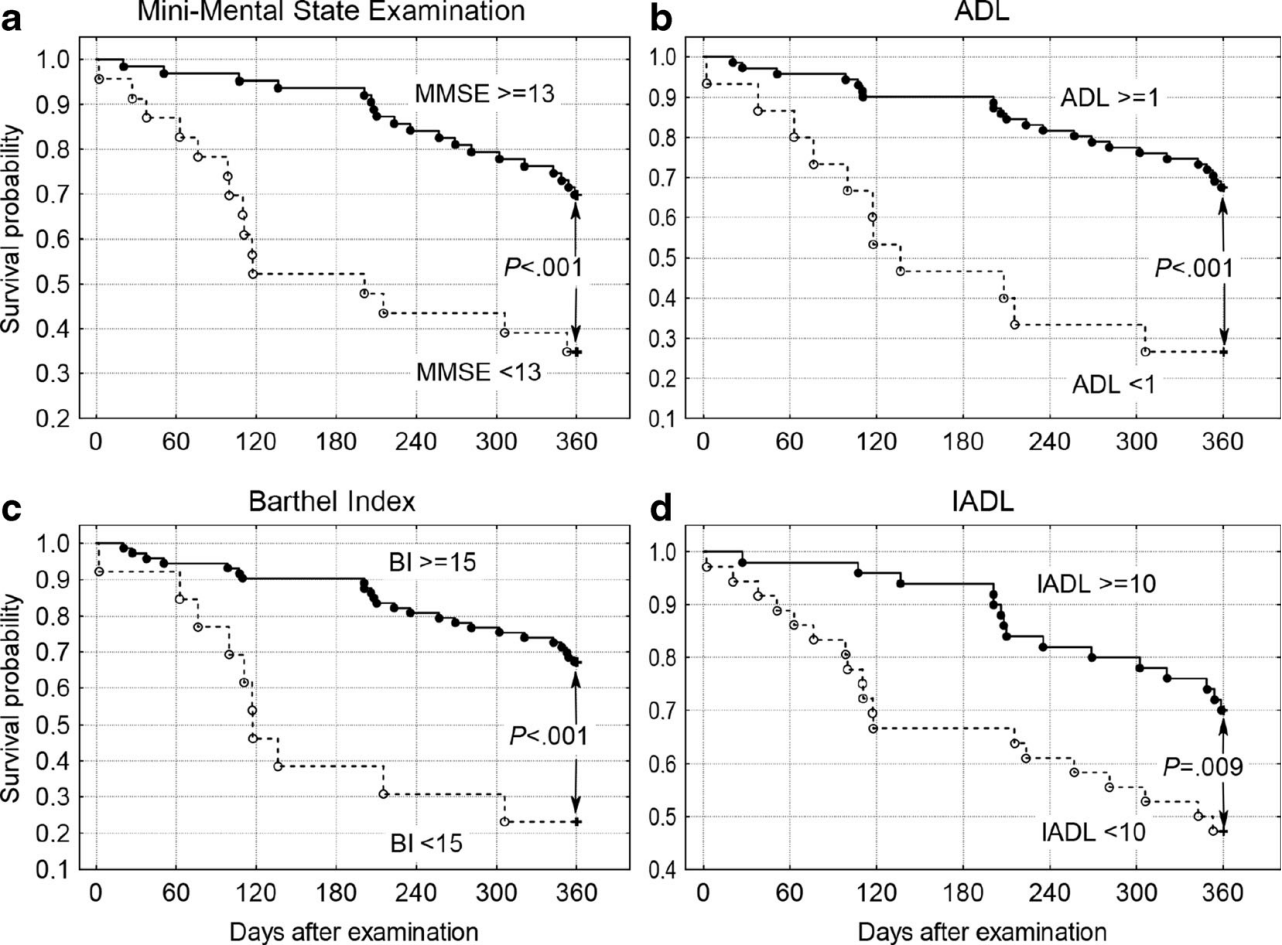
Kaplan–Meier survival probability of centenarians classified according to **a** Mini-Mental State Examination scores ≥13 versus lower values, **b** Katz Index of Independence in Activities of Daily Living (ADL) ≥1 versus lower values, **c** Barthel Index of Activities of Daily Living (Barthel Index) ≥15 versus lower values, and **d** Lawton Instrumental Activities of Daily Living Scale (IADL) ≥10 versus lower values

**Fig. 2:**
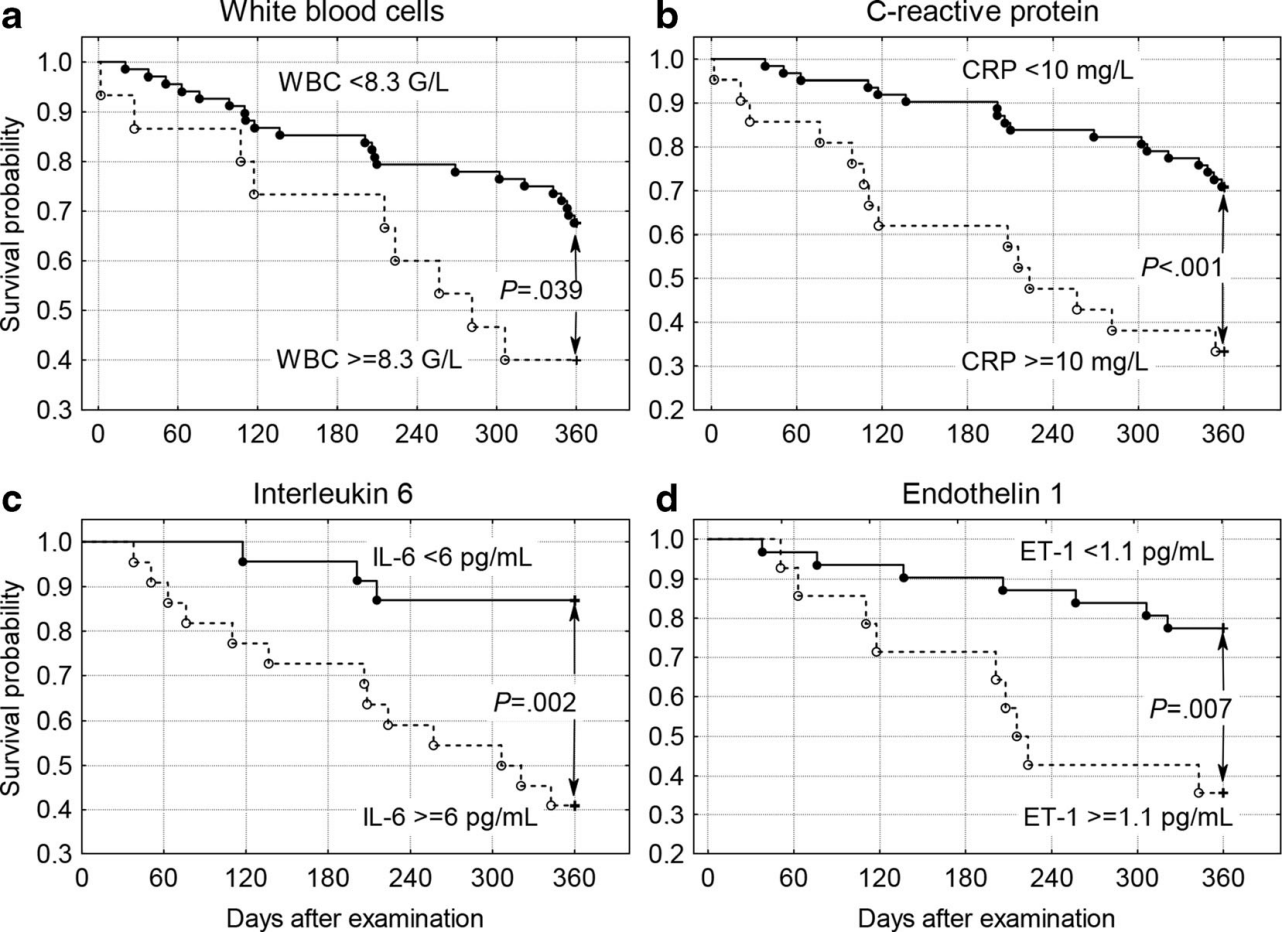
Kaplan–Meier survival probability of centenarians classified according to **a** white blood cells <8.3 G/L versus higher values, **b** C-reactive protein concentration <10 mg/L versus higher values, **c** interleukin-6 concentration <6 pg/mL versus higher values, and **d** endothelin-1 concentration <1.1 pg/mL versus higher values

Out of the 86 study participants, 52 (60 %) survived 360 days or more. Survivors compared to non-survivors were more likely to have hypertension; higher mean MMSE scores; higher serum albumin levels; and lower mean serum levels of CRP, IL-6, and ET-1 (Tables 1 and 2). The Mann–Whitney *U* test did not reveal significant differences between survivors and non-survivors regarding body mass index, blood pressure, and functional measures. In the univariate Cox proportional hazards model, improved centenarian survival was associated with a higher MMSE, Barthel Index, and serum albumin concentration. In addition, centenarian longevity was associated with a lower white blood cell (WBC) count as well as lower serum CRP, IL-6, and ET-1 concentrations (Table 3). We also found that centenarian survival was inversely correlated with IL-6 after adjustment for WBC, CRP, TNF-α, and ET-1 (hazard ratio, HR = 1.211, 95 % confidence interval (CI) 1.005–1.459; *P* < .044) and for ET-1 after adjustment for WBC, CRP, TNF-α, and IL-6 (HR = 4.412, 95 % CI 1.019–19.105; *P* < .047). Individuals with MMSE ≥13 points had higher 360-day survival probability (*P* < .001) as did subjects with Katz ADL ≥1 point (*P* < .001), Barthel Index ≥15 points (*P* < .001), and Lawton IADL ≥10 points (*P* = .009)  (Fig. 1). Higher probability of 360-day survival was also observed with WBC levels lower than 8.3 g/L (*P* = .039), CRP serum concentrations lower than 10 mg/L (*P* = .001), IL-6 lower than 6 pg/mL, and ET-1 lower than 1.1 pg/mL (Fig. 2). Centenarians, when compared with quinquagenarians, had higher concentrations of vascular activation markers (sP-selectin, sICAM-1, sVCAM-1), inflammatory markers (IL-6 and TNF-α), MMP-1, TIMP-1, and MMP-9 and lower sCD40-L and PAI-1 concentrations (Table 2). Spearman’s rank correlation analysis revealed a dependency between functional measures and certain biochemical markers (Table 4). ET-1 correlated neither with systolic nor with diastolic blood pressure or any marker of inflammation.

**Table 2:** Selected biochemical markers in the centenarian group (*n* = 43) and the quinquagenarian control group (*n* = 30)

Measure	Centenarian group				Quinquagenarian control group *n* = 30	Centenarians vs quinquagenarians *P* value
	All subjects *n* = 43	Survivors *n* = 28	Non-survivors *n* = 15	Survivors vs non-survivors *P* value		
Age, years	101.1 ± 1.0	100.9 ± 0.7	101.4 ± 1.3	.457	51.8 ± 5.0	<.001
sP-selectin, ng/mL	77.9 ± 86.5	84.0 ± 102.7	67.9 ± 49.9	.497	35.7 ± 17.4	<.001
sICAM-1, ng/mL	258 ± 114	257 ± 83	261 ± 154	.652	166 ± 77	<.001
sVCAM-1, ng/mL	689 ± 251	660 ± 218	737 ± 296	.373	428 ± 109	<.001
sCD40-L, pg/mL	795 ± 2330	906 ± 2880	672 ± 1110	.246	1690 ± 4649	.010
IL-6, pg/mL	5.92 ± 3.41	4.84 ± 3.41	7.87 ± 2.47	.003	1.03 ± 0.65	<.001
ET-1, pg/mL	1.03 ± 0.32	0.95 ± 0.29	1.19 ± 0.33	.005	0.92 ± 0.34	.150
TNF-α, pg/mL	4.13 ± 6.09	3.44 ± 4.65	5.39 ± 8.11	.407	1.00 ± 0.31	<.001
PAI-1, ng/mL	15.5 ± 5.0	19.4 ± 61.8	8.1 ± 5.9	.949	37.6 ± 72.4	.006
MMP-1, ng/mL	12.7 ± 49.5	15.7 ± 61.0	6.9 ± 1.3	.532	1.9 ± 49.6	<.001
TIMP-1, ng/mL	504 ± 166	485 ± 177	540 ± 141	.198	183 ± 74	<.001
MMP-9, ng/mL	1192 ± 762	1164 ± 737	1246 ± 830	.769	159 ± 96	<.001

**Table 3:** The Cox proportional hazards model univariate survival analysis for functional and biochemical measures (hazard ratio of death per unit of measure)

Measure	Hazard ratio	95 % confidence interval	*P* value
MMSE, points^a^	0.934	0.896–0.975	.002
Katz ADL, points^a^	0.840	0.716–0.985	.032
Barthel Index, points^a^	0.988	0.977–0.999	.026
Albumin, g/L^b^	0.926	0.870–0.986	.016
White blood cells, G/L^b^	1.161	1.059–1.273	.001
C-reactive protein, mg/L^b^	1.032	1.014–1.050	<.001
TNF-α^c^	1.024	0.960–1.091	.473
IL-6, pg/mL^c^	1.182	1.047–1.335	.007
ET-1, pg/mL^c^	3.711	1.233–11.169	.020

^a^Analysis of 86 centenarians, among them 52 survivors and 34 non-survivors

^b^Analysis of 84 centenarians, among them 52 survivors and 32 non-survivors

^c^Analysis of 43 centenarians, among them 28 survivors and 15 non-survivors

**Table 4:** Correlations between blood pressure, functional measures, and biochemical markers in centenarians (*n* = 43)

Functional measure	Biochemical marker	Spearman’s rank correlation coefficient	*P* value
Systolic blood pressure	ET-1	−0.031	.845
	IL-6	−0.371	.014
	PAI	−0.325	.034
Diastolic blood pressure	ET-1	−0.060	.702
	PAI	−0.345	.023
MMSE	Albumin	0.592	<.001
	CRP	−0.316	.041
	TNF-α	−0.318	.038
Katz ADL	Albumin	0.559	<.001
	CRP	−0.389	.011
	IL-6	−0.410	.006
Barthel Index	Albumin	0.563	<.001
	CRP	−0.381	.013
	IL-6	−0.355	.020
	TNF-α	−0.333	.029
Lawton IADL	Albumin	0.473	.001
	C-reactive protein	−0.482	.001
	sP-selectin	0.352	.021
	IL-6	−0.416	.006
	PAI-1	−0.340	.026
IL-6	CRP	0.406	.006
	TNF-α	0.390	.008
sP-selectin	MMP-1	0.359	.018
	MMP-9	0.560	<.001
sICAM-1	sVCAM-1	0.476	.001
	PAI-1	0.305	.047
sVCAM-1	sICAM-1	0.476	.001
	TIMP-1	0.313	.041
	sCD40-L	0.374	.015
sCD40-L	sVCAM-1	0.374	.015
	PAI-1	0.687	<.001
	MMP-1	0.326	.035
TIMP-1	MMP-9	0.387	.010

## Discussion

We conducted a multidimensional analysis of health status in a representative group of centenarians, including clinical, functional, and biochemical measures with respect to 360-day survival. We also determined cytokine levels in a control group of healthy quinquagenarians. Centenarians who survived 360 days, as compared with non-survivors, did not demonstrate significant differences in terms of initial clinical measures. These measures include significant medical conditions, current pharmacological treatment, functional status (aside from significant MMSE variation), and physical examination findings. Minimal variation in laboratory data was observed in our study group, with the exception of albumin which is a recognized predictor of survival in elderly patients (McMurtry and Rosenthal 1995; D’Erasmo et al. 1997) and CRP which is a risk factor for cardiovascular disease (Tracy et al. 1997; Buckley et al. 2009) (Table 1). Although blood pressure was not significantly higher in subjects who survived 360 days, hypertension was more common in the survivor group. Some differences that had been significant with regard to 180-day survival, e.g., systolic blood pressure, mean arterial pressure, basic and instrumental activities of daily living measures, creatinine, cystatin C, or folate serum levels (Szewieczek et al. 2015), lost their significance with respect to 360-day survival. However, Cox proportional hazards regression model (in univariate analyses) and Kaplan–Meier survival analysis combined with the Wilcoxon–Gehan statistic nonetheless suggest a predictive value not only of MMSE but also measures of independence in basic and instrumental activities of daily living (Katz ADL, Barthel Index, and Lawton IADL for 360-day survival) (Fig. 1, Table 3). Functional measures as a predictor of centenarian longevity is in agreement with results observed by others (Mossakowska et al. 2014). We found that among functional measures, MMSE scores are particularly potent predictors of survival in elderly populations (Park et al. 2013; Santabárbara et al. 2014) including centenarians (Mossakowska et al. 2014) and suggest that cognitive function reflects global health status in centenarians.

We examined endogenous factors to show an association, if any, with current functional status and longevity among centenarians. Our results revealed increased levels of inflammatory markers and/or vascular function markers including adhesion molecules, markers of extracellular matrix metabolism, and arterial stiffness (with the exception of sCD40-L, ET-1, and PAI-1) in centenarians as compared with healthy quinquagenarians (Table 2). We found also multiple correlations between these molecules (Table 4). These results confirmed the existence of low-grade systemic inflammation in our centenarian study group, in line with centenarian studies found in the literature (Franceschi et al. 2007). Richter et al. (2003) reported increased levels of adhesion molecules associated with age. Surprisingly, our results suggest that centenarians, compared with quinquagenarians, had lower PAI-1 levels (Table 2). These results seem to be inconsistent with the role of PAI-1 as a marker of age-related hypercoagulability (Cesari et al. 2010). Higher 360-day survival probability was associated with lower WBC and lower levels of CRP, IL-6, and ET-1 (Fig. 2). Finally, we observed positive correlations between albumin and functional measures and negative correlations between inflammatory markers and functional measures (Table 4). The association between survival and IL-6 was also significant after adjustment for WBC, CRP, TNF-α, and ET-1. Of note, IL-6 correlated negatively with systolic blood pressure (Table 4), while blood pressure was identified as a factor positively correlated with 180-day survival in centenarians (Szewieczek et al. 2015). These results indicate the significant influence of the inflammatory state on cognitive function and independence in basic and instrumental activities of daily living. Blüher et al. (2005) reported on the relationship between CRP and IL-6 and impaired glucose tolerance, as well as between IL-6 and obesity, glucose metabolism, and insulin resistance. An association between elevated levels of TNF-α (but not IL-6) and mortality in a group of 126 centenarians was observed by Bruunsgaard et al. (2003). CRP was not associated with increased mortality when TNF-α was included in the analysis. Inconsistent results were obtained in a large population-based cohort (Wichmann et al. 2014), where elevated but constant CRP was associated with lower risk of cognitive impairment while an increasing CRP level over a period of 20 years was associated with increased risk of cognitive impairment in statin nonusers. In addition, both high IL-6 and its increase were associated with greater risk of cognitive impairment. Centenarians who survived 180 days had lower sVCAM-1 levels as compared with those who did not survive (Szewieczek et al. 2011). However, the results of our study suggest that there was no correlation between centenarian 360-day survival and vascular status markers sP-selectin, sICAM-1, and sVCAM-1, with the exception of ET-1. Despite a lack of correlation between 360-day survival and vascular status markers, there was a positive correlation between inflammatory markers and vascular status makers. These vascular status markers were markedly higher in centenarians in comparison to younger individuals. Their concentration depends on TNF-α and IL-6, but many other factors can also influence their production (Wojakowski and Gminski 2001; Blann et al. 2003). High levels of circulating adhesion molecules are considered independent risk factors for cardiovascular diseases (Hwang et al. 1997). Simultaneous measurement of sICAM-1 and CRP may serve as a risk marker for future coronary events (Luc et al. 2003). Among patients with coronary artery disease, soluble adhesion molecules sVCAM-1 and sICAM-1 are risk markers for death due to cardiovascular complications (Blankenberg et al. 2001). Markers of arterial stiffness and extracellular matrix metabolism (MMP-1, MMP-9, and TIMP-1) were also markedly higher in centenarians as compared with quinquagenarians, indicating higher cardiovascular risk (Bizbiz et al. 1996). MMP-9 and TIMP-1 were found to be related to all-cause mortality risk in elderly patients (Hansson et al. 2011). Soluble CD40L is another marker of immunological activity. CD40 and CD40 ligand (CD40L) have a critical role in the pathophysiology of atherosclerosis and atherothrombosis (Yuan et al. 2010). We observed lower sCD40L levels in centenarians as compared to quinquagenarians (Table 2). However, sCD40L levels correlated positively with sVCAM-1, MMP-1, and PAI-1. Our earlier paper revealed higher sCD40L levels in centenarians who survived 180 days as compared with non-survivors (Szewieczek et al. 2011) (Table 2). Our results seem to be inconsistent with results observed in other studies. In geriatric study groups, higher sCD40L levels were observed than in younger study groups, although geriatric patient groups under the age of 80 years had higher sCD40L levels than geriatric patient groups over the age of 80 in the same study (Inami et al. 2009). Baldus et al. (2003) observed an association between sCD40L and increased cardiac risk in patients with acute coronary syndromes in the CAPTURE trial. Soluble CD40L is considered a risk marker both for cardiovascular disease and adverse events, especially in patients with acute coronary disease (Tousoulis et al. 2010). An association between increased levels of sCD40L and metabolic syndrome in prediabetic patients, hypertension, and elevated cholesterol concentration has been reported (Genc et al. 2012). However, in TACTICS-TIMI 18, sCD40L was not associated with an increased risk of ischemic events in non-ST elevation acute coronary syndromes when patients were treated with platelet GPIIb/IIIa inhibitors (Morrow et al. 2008). Similarly, no association between sCD40L and cardiovascular outcomes in a large cohort of patients with acute coronary syndrome in OPUS TIMI-16 trial was found (Olenchock et al. 2008). Thus, the role of sCD40L as a cardiovascular risk marker needs further evaluation. A novel finding of our study was identification of potent vasoconstrictor ET-1 as a negative survival risk marker in centenarians. ET-1 is involved in the pathogenesis of hypertension, atherosclerosis, and chronic heart failure (Brunner et al. 2006). It has been revealed that ET-1 exerts proinflammatory activity (Elisa et al. 2015). We found a distinct association between ET-1 levels and mortality in centenarians. ET-1 levels of 1.1 pg/mL or more were associated with a 54 % reduction in 360-day survival probability (Fig. 2). The relationship between ET-1 and 360-day survival was still significant after adjustment for WBC, CRP, TNF-α, and IL-6. ET-1 was already identified as a strong independent predictor of increased mortality in elderly patients with severe congestive heart failure (Selvais et al. 2000; Van Beneden et al. 2004). However, our observation might be surprising because we had previously identified mildly elevated blood pressure to be a factor associated with higher 180-day survival probability in the same group of centenarians (Szewieczek et al. 2015). In other studies, ET-1 correlated positively with systolic blood pressure in septuagenarians treated for hypertension (Skalska et al. 2009). In our study, ET-1 correlated neither with blood pressure in centenarians (Table 4) nor with any inflammatory markers that might suggest that the vasoconstrictive action of this molecule can be diminished with very advanced age.

A major limitation of our study was the relatively low number of subjects of whom only half had performed all biochemical tests, which may influence statistical analysis.

In summary, our results indicate that the phenomenon described as inflammaging not only exists in centenarians but also influences their performance and survival. Functional measures, especially MMSE, and inflammatory markers as well as some vascular function molecules can be considered prognostic survival factors; among them, IL-6 and ET-1 revealed the most potent significance as independent survival risk markers in centenarians. For centenarians, the prognostic role of traditional metabolic indices like glucose, cholesterol, and creatinine levels are significantly limited.

## Conclusion

Functional level is associated with an inflammatory state in centenarians; functional measures, inflammatory markers, and endothelin-1 are predictors of 360-day survival in centenarians.

## References

[CR1] Andersen SL, Sebastiani P, Dworkis DA, Feldman L, Perls TT (2012). Health span approximates life span among many supercentenarians: compression of morbidity at the approximate limit of life span. J Gerontol A Biol Sci Med Sci.

[CR2] Arnold J, Dai J, Nahapetyan L, Arte A, Johnson MA, Hausman D, Rodgers WL, Hensley R, Martin P, Macdonald M, Davey A, Siegler IC, Jazwinski SM, Poon LW (2010). Predicting successful aging in a population-based sample of Georgia centenarians. Curr Gerontol Geriatr Res.

[CR3] Baldus S, Heeschen C, Meinertz T, Zeiher AM, Eiserich JP, Münzel T, Simoons ML, Hamm CW, CAPTURE investigators (2003). Myeloperoxidase serum levels predict risk in patients with acute coronary syndromes. Circulation.

[CR4] Barodka VM, Joshi BL, Berkowitz DE, Hogue CW, Nyhan D (2011). Review article: implications of vascular aging. Anesth Analg.

[CR5] Bizbiz L, Bonithon-Kopp C, Ducimetierè P, Berr C, Alperovitch A, Robert L (1996). Relation of serum elastase activity to ultrasonographically assessed carotid artery wall lesions and cardiovascular risk factors. The EVA study. Atherosclerosis.

[CR6] Blankenberg S, Rupprecht HJ, Bickel C, Peetz D, Hafner G, Tiret L, Meyer J (2001). Circulating cell adhesion molecules and death in patients with coronary artery disease. Circulation.

[CR7] Blann AD, Nadar SK, Lip GY (2003). The adhesion molecule P-selectin and cardiovascular disease. Eur Heart J.

[CR8] Blüher M, Fasshauer M, Tönjes A, Kratzsch J, Schön MR, Paschke R (2005). Association of interleukin-6, C-reactive protein, interleukin-10 and adiponectin plasma concentrations with measures of obesity, insulin sensitivity and glucose metabolism. Exp Clin Endocrinol Diabetes.

[CR9] Brunner F, Brás-Silva C, Cerdeira AS, Leite-Moreira AF (2006). Cardiovascular endothelins: essential regulators of cardiovascular homeostasis. Pharmacol Ther.

[CR10] Bruunsgaard H, Andersen-Ranberg K, Hjelmborg JvB, Pedersen BK, Jeune B (2003). Elevated levels of tumor necrosis factor alpha and mortality in centenarians. Am J Med.

[CR11] Buckley DI, Fu R, Freeman M, Rogers K, Helfand M (2009). C-reactive protein as a risk factor for coronary heart disease: a systematic review and meta-analyses for the U.S. Preventive Services Task Force. Ann Intern Med.

[CR12] Central Statistical Office of Poland (2014) Prognoza ludnosci na lata 2014-2050 (opracowana 2014 r.) http://stat.gov.pl/obszary-tematyczne/ludnosc/prognoza-ludnosci/prognoza-ludnosci-na-lata-2014-2050-opracowana-2014-r-,1,5.html. Accessed 28 March 2015

[CR13] Cesari M, Penninx BW, Newman AB, Kritchevsky SB, Nicklas BJ, Sutton-Tyrrell K, Rubin SM, Ding J, Simonsick EM, Harris TB, Pahor M (2003). Inflammatory markers and onset of cardiovascular events: results from the Health ABC study. Circulation.

[CR14] Cesari M, Pahor M, Incalzi RA (2010). Plasminogen activator inhibitor-1 (PAI-1): a key factor linking fibrinolysis and age-related subclinical and clinical conditions. Cardiovasc Ther.

[CR15] Cunningham C, Hennessy E (2015). Co-morbidity and systemic inflammation as drivers of cognitive decline: new experimental models adopting a broader paradigm in dementia research. Alzheimers Res Ther.

[CR16] D’Erasmo E, Pisani D, Ragno A, Romagnoli S, Spagna G, Acca M (1997). Serum albumin level at admission: mortality and clinical outcome in geriatric patients. Am J Med Sci.

[CR17] Elisa T, Antonio P, Giuseppe P, Alessandro B, Giuseppe A, Federico C, Marzia D, Ruggero B, Giacomo M, Andrea O, Daniela R, Mariaelisa R, Claudio L (2015). Endothelin receptors expressed by immune cells are involved in modulation of inflammation and in fibrosis: relevance to the pathogenesis of systemic sclerosis. J Immunol Res.

[CR18] Evert J, Lawler E, Bogan H, Perls T (2003). Morbidity profiles of centenarians: survivors, delayers and escapers. J Gerontol A Biol Sci Med Sci.

[CR19] Folstein MF, Folstein SE, McHugh PR (1975). Mini-Mental State: a practical method for grading the cognitive state of patients for the clinician. J Psychiatr Res.

[CR20] Franceschi C, Bonafè M (2003). Centenarians as a model for healthy aging. Biochem Soc Trans.

[CR21] Franceschi C, Campisi J (2014). Chronic inflammation (inflammaging) and its potential contribution to age-associated diseases. J Gerontol A Biol Sci Med Sci.

[CR22] Franceschi C, Bonafè M, Valensin S, Olivieri F, De Luca M, Ottaviani E, De Benedictis G (2000). Inflamm-aging. An evolutionary perspective on immunosenescence. Ann N Y Acad Sci.

[CR23] Franceschi C, Capri M, Monti D, Giunta S, Olivieri F, Sevini F, Panourgia MP, Invidia L, Celani L, Scurti M, Cevenini E, Castellani GC, Salvioli S (2007). Inflammaging and anti-inflammaging: a systemic perspective on aging and longevity emerged from studies in humans. Mech Ageing Dev.

[CR24] Genc H, Dogru T, Tapan S, Tasci I, Bozoglu E, Gok M, Aslan F, Celebi G, Erdem G, Avcu F, Ural AU, Sonmez A (2012). Soluble CD40 ligand, soluble P-selectin and von Willebrand factor levels in subjects with prediabetes: the impact of metabolic syndrome. Clin Biochem.

[CR25] Hansson J, Vasan RS, Ärnlöv J, Ingelsson E, Lind L, Larsson A, Michaëlsson K, Sundström J (2011). Biomarkers of extracellular matrix metabolism (MMP-9 and TIMP-1) and risk of stroke, myocardial infarction, and cause-specific mortality: cohort study. PLoS One.

[CR26] Hwang SJ, Ballantyne CM, Sharrett AR, Smith LC, Davis CE, Gotto AM, Boerwinkle E (1997). Circulating adhesion molecules VCAM-1, ICAM-1, and E-selectin in carotid atherosclerosis and incident coronary heart disease cases: the Atherosclerosis Risk In Communities (ARIC) study. Circulation.

[CR27] Inami N, Nomura S, Inami O, Kimura Y, Urase F, Maeda Y, Iwasaka T (2009). Significance of soluble CD40 ligand, adiponectin and reactive oxygen metabolites in aging. Arch Gerontol Geriatr.

[CR28] Katz S, Ford AB, Moskowitz RW, Jackson BA, Jaffe MW (1963). Studies of illness in the aged. The index of ADL: a standardized measure of biological and psychosocial function. JAMA.

[CR29] Lawton MP, Brody EM (1969). Assessment of older people: self-maintaining and instrumental activities of daily living. Gerontologist.

[CR30] Luc G, Arveiler D, Evans A, Amouyel P, Ferrieres J, Bard JM, Elkhalil L, Fruchart JC, Ducimetiere P, PRIME Study Group (2003). Circulating soluble adhesion molecules ICAM-1 and VCAM-1 and incident coronary heart disease: the PRIME Study. Atherosclerosis.

[CR31] Maes M, DeVos N, Wauters A, Demedts P, Maurits VW, Neels H, Bosmans E, Altamura C, Lin A, Song C, Vandenbroucke M, Scharpe S (1999). Inflammatory markers in younger vs elderly normal volunteers and in patients with Alzheimer’s disease. J Psychiatr Res.

[CR32] Mahoney FI, Barthel DW (1965). Functional evaluation: the Barthel Index. Md State Med J.

[CR33] McMurtry CT, Rosenthal A (1995). Predictors of 2-year mortality among older male veterans on a geriatric rehabilitation unit. J Am Geriatr Soc.

[CR34] Michaud M, Balardy L, Moulis G, Gaudin C, Peyrot C, Vellas B, Cesari M, Nourhashemi F (2013). Proinflammatory cytokines, aging, and age-related diseases. J Am Med Dir Assoc.

[CR35] Morrow DA, Sabatine MS, Brennan ML, de Lemos JA, Murphy SA, Ruff CT, Rifai N, Cannon CP, Hazen SL (2008). Concurrent evaluation of novel cardiac biomarkers in acute coronary syndrome: myeloperoxidase and soluble CD40 ligand and the risk of recurrent ischaemic events in TACTICS-TIMI 18. Eur Heart J.

[CR36] Mossakowska M, Broczek K, Wieczorowska-Tobis K, Klich-Rączka A, Jonas M, Pawlik-Pachucka E, Safranow K, Kuznicki J, Puzianowska-Kuznicka M (2014). Cognitive performance and functional status are the major factors predicting survival of centenarians in Poland. J Gerontol A Biol Sci Med Sci.

[CR37] Olenchock BA, Wiviott SD, Murphy SA, Cannon CP, Rifai N, Braunwald E, Morrow DA (2008). Lack of association between soluble CD40L and risk in a large cohort of patients with acute coronary syndrome in OPUS TIMI-16. J Thromb Thrombolysis.

[CR38] Park MH, Kwon DY, Jung JM, Han C, Jo I, Jo SA (2013). Mini-Mental Status Examination as predictors of mortality in the elderly. Acta Psychiatr Scand.

[CR39] Paulus P, Jennewein C, Zacharowski K (2011). Biomarkers of endothelial dysfunction: can they help us deciphering systemic inflammation and sepsis?. Biomarkers.

[CR40] Richter V, Rassoul F, Purschwitz K, Hentschel B, Reuter W, Kuntze T (2003). Circulating vascular cell adhesion molecules VCAM-1, ICAM-1, and E-selectin in dependence on aging. Gerontology.

[CR41] Santabárbara J, Lopez-Anton R, Marcos G, De-la-Cámara C, Lobo E, Saz P, Gracia-García P, Ventura T, Campayo A, Rodríguez-Mañas L, Olaya B, Haro JM, Salvador-Carulla L, Sartorius N, Lobo A (2014). Degree of cognitive impairment and mortality: a 17-year follow-up in a community study. Epidemiol Psychiatr Sci.

[CR42] Sebastiani P, Sun FX, Andersen SL, Lee JH, Wojczynski MK, Sanders JL, Yashin A, Newman AB, Perls TT (2013). Families Enriched for Exceptional Longevity also have Increased Health-Span: Findings from the Long Life Family Study. Front Public Health.

[CR43] Selvais PL, Robert A, Ahn S, van Linden F, Ketelslegers JM, Pouleur H, Rousseau MF (2000). Direct comparison between endothelin-1, N-terminal proatrial natriuretic factor, and brain natriuretic peptide as prognostic markers of survival in congestive heart failure. J Card Fail.

[CR44] Skalska AB, Pietrzycka A, Stepniewski M (2009). Correlation of endothelin-1 plasma levels with plasma antioxidant capacity in elderly patients treated for hypertension. Clin Biochem.

[CR45] Sun Z (2015). Aging, arterial stiffness, and hypertension. Hypertension.

[CR46] Szewieczek J, Dulawa J, Gminski J, Kurek A, Legierska K, Francuz T, Włodarczyk-Sporek I, Janusz-Jenczen M, Hornik B (2011). Better cognitive and physical performance is associated with higher blood pressure in centenarians. J Nutr Health Aging.

[CR47] Szewieczek J, Dulawa J, Francuz T, Legierska K, Hornik B, Włodarczyk-Sporek I, Janusz-Jenczeń M, Batko-Szwaczka A (2015). Mildly elevated blood pressure is a marker for better health status in Polish centenarians. Age.

[CR48] Tan ZS, Beiser AS, Vasan RS, Roubenoff R, Dinarello CA, Harris TB, Benjamin EJ, Au R, Kiel DP, Wolf PA, Seshadri S (2007). Inflammatory markers and the risk of Alzheimer disease: the Framingham Study. Neurology.

[CR49] Terry DF, Sebastiani P, Andersen SL, Perls TT (2008). Disentangling the roles of disability and morbidity in survival to exceptional old age. Arch Intern Med.

[CR50] Thakore AH, Guo CY, Larson MG, Corey D, Wang TJ, Vasan RS, D’Agostino RB, Lipinska I, Keaney JF, Benjamin EJ, O’Donnell CJ (2007). Association of multiple inflammatory markers with carotid intimal medial thickness and stenosis (from the Framingham Heart Study). Am J Cardiol.

[CR51] Tousoulis D, Androulakis E, Papageorgiou N, Briasoulis A, Siasos G, Antoniades C, Stefanadis C (2010). From atherosclerosis to acute coronary syndromes: the role of soluble CD40 ligand. Trends Cardiovasc Med.

[CR52] Tracy RP, Lemaitre RN, Psaty BM, Ives DG, Evans RW, Cushman M, Meilahn EN, Kuller LH (1997). Relationship of C-reactive protein to risk of cardiovascular disease in the elderly. Results from the Cardiovascular Health Study and the Rural Health Promotion Project. Arterioscler Thromb Vasc Biol.

[CR53] Van Beneden R, Gurné O, Selvais PL, Ahn SA, Robert AR, Ketelslegers JM, Pouleur HG, Rousseau MF (2004). Superiority of big endothelin-1 and endothelin-1 over natriuretic peptides in predicting survival in severe congestive heart failure: a 7-year follow-up study. J Card Fail.

[CR54] Wichmann MA, Cruickshanks KJ, Carlsson CM, Chappell R, Fischer ME, Klein BE, Klein R, Tsai MY, Schubert CR (2014). Long-term systemic inflammation and cognitive impairment in a population-based cohort. J Am Geriatr Soc.

[CR55] Willcox DC, Willcox BJ, Poon LW (2010). Centenarian studies: important contributors to our understanding of the aging process and longevity. Curr Gerontol Geriatr Res.

[CR56] Wojakowski W, Gminski J (2001). Soluble ICAM-1, VCAM-1 and E-selectin in children from families with high risk of atherosclerosis. Int J Mol Med.

[CR57] Yuan M, Ohishi M, Wang L, Raguki H, Wang H, Tao L, Ren J (2010). Association between serum levels of soluble CD40/CD40 ligand and organ damage in hypertensive patients. Clin Exp Pharmacol Physiol.

[CR58] Zakynthinos E, Pappa N (2009). Inflammatory biomarkers in coronary artery disease. J Cardiol.

